# Emergency Responses to Covid-19 Outbreak: Experiences and Lessons from a General Hospital in Nanjing, China

**DOI:** 10.1007/s00270-020-02474-w

**Published:** 2020-04-27

**Authors:** Yang Shen, Ying Cui, Ning Li, Chen Tian, Ming Chen, Ye-Wei Zhang, Ying-Zi Huang, Hui Chen, Qing-Fang Kong, Qun Zhang, Gao-Jun Teng

**Affiliations:** 1 Department of Obstetrics and Gynecology, Zhongda Hospital, Southeast University, Nanjing, Jiangsu Province China; 2 Center of Interventional Radiology and Vascular Surgery, Department of Radiology, Zhongda Hospital, Southeast University, 87 Dingjiaqiao Rd., Nanjing, Jiangsu Province China; 3 Department of Neurosurgery, Zhongda Hospital, Southeast University, Nanjing, Jiangsu Province China; 4 Department of Infectious Diseases, Zhongda Hospital, Southeast University, Nanjing, Jiangsu Province China; 5 Department of Urology, Zhongda Hospital, Southeast University, Nanjing, Jiangsu Province China; 6 Department of Medical Affairs, Zhongda Hospital, Southeast University, Nanjing, Jiangsu Province China; 7 Department of Critical Care Medicine, Zhongda Hospital, Southeast University, Nanjing, Jiangsu Province China; 8 Department of Infection Prevention and Control, Zhongda Hospital, Southeast University, Nanjing, Jiangsu Province China

**Keywords:** Covid-19, Hospital management, Infection prevention and control, Emergency response

## Abstract

**Background:**

The novel coronavirus 2019 (SARS-CoV-2) has caused wide dissemination across the world. Global health systems are facing the unprecedented challenges. Here we shared the experiences and lessons in emergency responses and management from our hospital, a government-assigned regional anti-Covid-19 general hospital in Nanjing, Jiangsu Province, China.

**Methods:**

Our periodic strategies in dealing with Covid-19 were described in detail. An administrative response including the establishment of Emergency Leadership Committee that was in full charge of management was established. Modifications of infrastructure including the Fever Clinic, inpatient ward, outpatient clinic and operation room were carried out. Special arrangements for outpatient services, hospitalization and surgeries were introduced. Medical personnel training and patient educations were performed. Initiations of Covid-19 researches and application of information technology were introduced.

**Findings:**

Since January 16, three cases have been confirmed in our hospital and no healthcare-associated infection was found. During the epidemics, 6.46% staffs suffered depression, 9.87% had anxiety, and 98% were satisfied with the infection control policy. Shortages in staffs and medical consumables, and limitation in space were the obstacles we encountered.

**Interpretation:**

As the cost of in-hospital transmission is unbearable, our experiences and lessons suggested that prompt actions should be taken immediately to decrease or eliminate potential in-hospital transmission. Experience shared herein may be useful for those facilities that are and may encounter Covid-19.

## Introduction

In late December 2019, the first case of the unknown resource of Novel Coronavirus was reported in Wuhan, China [1]. It is officially recognized as SARS-CoV-2 by the International Committee on Taxonomy of Viruses recently [2]. Being highly contagious, Covid-19 has arisen a national pandemic with more than 81,300 confirmed cases and 3248 deaths in China, and a rapid transmission globally to 166 countries worldwide [3]. As a consequence, WHO escalated risk assessment to “very high” at a global level on February 28. Without doubt, local and global health systems are facing unprecedented challenges in urgent need of the preparedness and proactive strategy to enhance screening, treatment and prevention of the infections.

Zhongda Hospital, Southeast University is an academic and comprehensive regional medical center in Nanjing, Jiangsu Province. It is equipped with 2000 beds and has 2604 employees and approximately 1000 interns, residents and fellows, serving 4000–5000 outpatients daily. Nanjing is a metropolis with population eight million located in the East China. Both lying on the bank of Yangtzi River, it has close linkage with Wuhan in economics and transportation. Nanjing was therefore assessed as a high-risk region by the government, with a total of 93 Covid-19 patients confirmed so far. Adapted to the governmental emergency response, Zhongda Hospital was officially appointed as one of the four referring hospitals for Covid-19 patients’ care in the city.

Confronting the outbreak and the ever-changing situation, we rapidly reacted and established periodic strategies including administrative team establishment, infrastructure modifications and special arrangement for clinic patients, hospitalizations and surgeries. Medical personnel training and patient educations were promoted, and advanced information technology was applied. We believe that these responses made significant contributions to the successful control of Covid-19 in-hospital spread while maintaining essential medical services. In the current paper, we would like to share our experiences and lessons in preparing and managements in the past nine weeks. It should be useful for the administration board, infection control team and any healthcare personnel who are facing or will encounter Covid-19.

## Methods and Measures

### Overview of Periodical Strategies

**Fig. 1:**
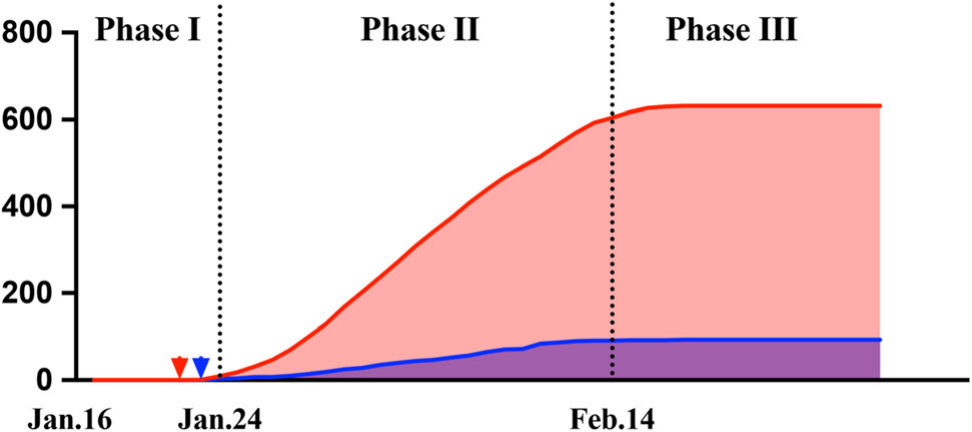
Three major phases of hospital emergency management. The first confirmed case in Jiangsu Province was reported on January 22 in Suzhou (red arrowhead), and the first patient in Nanjing was documented on the following day (blue arrowhead)

In general, the emergency responses of our hospital were sequentially practiced in three major phases according to the Covid-19 situation in Nanjing and China (Fig. 1) (Table 1).

**Table 1:** Overview of the three phases and periodical strategies

	Duration	China	Outside of China	Key measures in Zhongda Hospital
Phase I	January 16 to January 23	Declaration of human-to-human transmission 600 cases nationwide with substantial increase Covid-19 was ranked as Cat. B in China Wuhan locked down the city Nanjing’s first confirmed case reported on Jan 23	First case in the USA, Japan, Thailand, Vietnam, etc. Warnings for traveling and contacting Wuhan and China	Emergency Leadership Committee and advanced IPC and MDT establishment PPE and medical consumables reservation and preparation Representative protocols for Covid-19 cases and regular medical services Covid-19 education and training for physicians, nurses and hospital staffs Infrastructure modifications including the ward, Fever Clinic, quarantine unit and operating theater
Phase II	January 24 to January 14	Dramatic accumulation with more than 10,000 daily increase in confirmed and suspected cases in China Level I emergency status declaration in multiple cities Intercity traffic and transportation suspended Severe shortage in medical supplies	WHO determined a Public Health Emergency of International Concern International traffic restriction on China announced by 130 countries and regions (as of Feb 13)	Strict in-hospital flow control, temperature and Covid-19 RT-PCR screening covered 100% visitors and patients Enhanced personnel support to the Fever Clinic, Emergency and respiratory department Temporary suspension of elective surgeries, and special arrangement for emergency operation Attempts of online medical services and consultation
Phase III	Since February 15	Pandemic in China was under gradual control except Hubei Province New challenges from social and industrial production recovery, and imported infections emerges In Nanjing, a total of 93 Covid-19 cases were reported with no new case in 12 successive days (as of Mar 1)	Global spread in 58 countries with outpaced number than China (as of Mar 1) Worldwide anxiety affected social and financial system	All measures above continued in force Resumption of elective services under full monitoring Surgical and hospitalization workflow was individualized upon MDT evaluation and committee approval

Phase I: It was initially launched on January 16, at the time of the declaration of its potential for human-to-human transmission by Professor Nanshan Zhong, the appointed leading advisor in the epidemics. The number of confirmed cases quickly increased to 600 nationwide, with a continuous spreading potential, and thus Chinese government soon officially ranked Covid-19 as category B infectious disease, which was managed as category A. Structural and political alteration and improvement was our essential content in Phase I under the guidance of WHO and Centers for Disease Control and Prevention (CDC). These efforts in Phase I acted as a fundamental footstone throughout the time.

Phase II: With a progressive national outbreak in China and unfortunately an increasing number of confirmed cases in other countries, the WHO-convened second meeting of the Emergency Committee decided on the determination of a Public Health Emergency of International Concern. The heavy Chinese New Year travel further challenged the healthcare facilities. All preliminary measures that were taken in Phase I were immediately upgraded since the first case reported in Jiangsu and Nanjing on January 22 and 23 sequentially.

Phase III: With a prospection of the first wave of population movement after the national holiday, Phase II covered the entire Chinese New Year week and 14-day observational isolation until February 14. In China, Covid-19 in the regions other than Hubei Province showed a substantial control. However, the possibility of imported infection and transmission was higher than before. On the other hand, we were aware of the accumulation of patients waiting, especially those with semi-elective diseases, and the necessity for work resumption. Therefore, in Phase III from February 14, consultant clinic gradually reopened, and elective operations were allowed only for Covid-19-negative patients, while all the previous protocols still being effective.

### Administrative Response

**Fig. 2:**
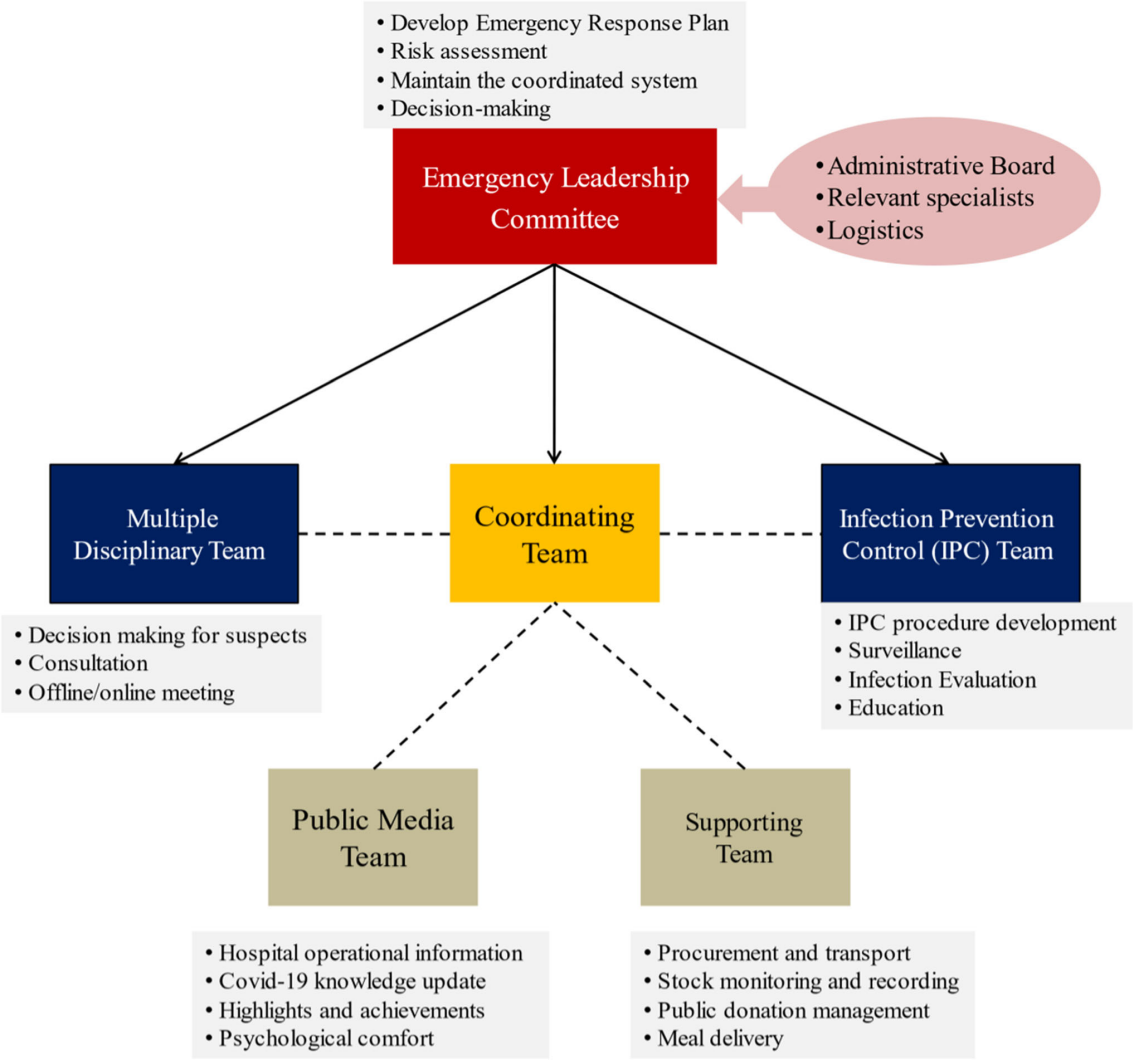
The organizational structure of the board

Emergency Leadership Committee—As a quick response to the outbreak of SARS-CoV-2, the Emergency Leadership Committee was launched on January 21. The committee, chaired by the President of Zhongda Hospital, included representatives from Administrative Board, relevant specialists, and logistics. This is the headquarter in responsibility for risk-assessment, decision-making and coordinating for the preparedness. An Emergency Response Plan was also established to clarify the responsibilities of each department to deal with potential internal and external emergencies. Figure 2 shows the organizational structure of the board. The Supporting Team and the Media Team were directly led by the Coordinating Team.

The Committee consists of: (1) Coordinating Team, to ensure the reallocation of personnel and resources for the potential large demand, responsible for the communication within the hospital, and with other healthcare institutions and governmental health authorities. (2) Multiple Disciplinary Team (MDT), to facilitate the quick decision making for suspected patients through either offline or online (web conference) consultation. It is composed of qualified experts from Emergency, Critical Care Medicine, Infection Prevention and Control, Respiratory, Laboratory services, and Medical Imaging. (3) Infection Prevention Control (IPC) Team, based on a specified policy. IPC team works to prevent nosocomial infections through enhanced surveillance, comprehensive evaluation, development of tailored procedures for each department, staff education, medical waste management, etc. (4) Supporting Team, to support the activities including procurement, transport, warehousing, stock monitoring and recording. Experiencing from the material shortage in Phase II, the access for public donation was immediately opened. All donations were strictly approved and were carefully examined to be qualified. (5) Public Media Team, to release daily real-time information through our own social media account, including knowledge update, operational information, highlights and achievements, psychological comfort, etc. The rapid release plays an important role in educating the public, avoiding patients' gathering, reducing the chance of in-hospital cross-infection and relieving social anxiety.

### Infrastructure Modifications

Fever Clinic—“Fever Clinic” is specifically designed as the first pass for the suspected outpatients who visit hospital with fever. The ventilation system was reorganized to prevent air backflow in the Fever Clinic. Contaminated (meeting patients), buffer corridor (donning and removing PPE) and hygiene (resting) blocks were separated. Two single-way inter-block paths were clapboarded, in such a way as to avoid the spread of pathogens efficiently. An isolated pedestrian connecting the Fever Clinic and quarantine unit was set.

**Fig. 3:**
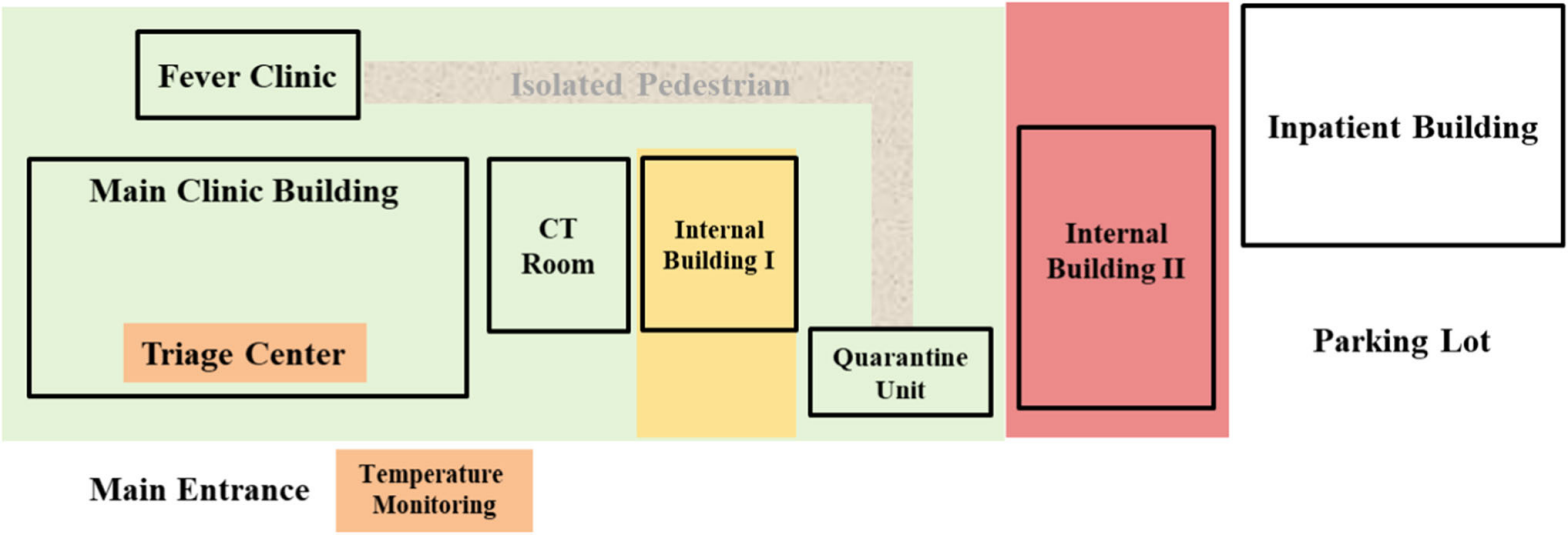
The illustration of the infrastructure modifications. The internal building in the figure indicates the building for in-patient in internal medicine unit

Quarantine Unit—Any individual with fever, chest CT abnormalities, or epidemic contact with Covid-19 were guided to a remote quarantined unit (Fig. 3, green area). This unit was remolded from a regular day-care unit, with an enhancement in isolation, ventilation and sterilization. We estimated an outbreak with a similar mass of patients and suspects in Nanjing, as such current quarantine ward would be in significant shortage. For this, additional spaces (Fig. 3, yellow and red areas) were prepared for backups if needed. The internal building in the figure refers to the building only for in-patients in internal medicine unit.

Emergency Department and Outpatient Clinic—We re-allocated the Department of Emergency and Respiratory and doubled the areas. A case with respiratory symptoms such as coughing, with or without sputum, but no fever can be instantly evaluated by a respiratory professional. The triage center takes full responsibility to collect the epidemiological history of every one of the visitors before entering the clinics. Though the senior physician clinics were temporarily closed to avoid extensive saturation of outpatients, regular clinic was guaranteed. Online consultation and prescription system were particularly recommended for non-emergencies.

Inpatient Ward—Ward facilities and capacities were thoroughly assessed and maintained. Prominent description of Covid-19 with infection indications and notice for mask-wearing and hand hygiene were posted. For cases for semi-elective surgeries such as malignant tumors, an isolation section with single-bed rooms was prepared.

Post-operational quarantine is granted until SARS-CoV-2 tested negative. This policy is still in effect for elective and semi-elective patients, despite that Covid-19 was relatively under control recently.

Laboratory and Medical Imaging—Clinical Laboratory and Radiology worked closely for SARS-CoV-2 screening. During the initial period, virus RT-PCR test was only provided by Jiangsu CDC. In Phase II, a RT-PCR for the virus in biosafety level two modification in the hospital was quickly set up to meet the subsequent national outbreak, complying with national and WHO Laboratory Biosafety Manual [4]. Collaboratively, an isolated CT room near the Fever Clinic and Emergency was assigned, in such a way to control the in-hospital transfer and thus reduce transmission risks (Fig. 3). Air sterilization with ultraviolet light for 10 min and medical sheets were replaced after each scanning.

Operating Room—In principle, surgeries were performed in a positive pressure operating theatre, which has been proved protective to staff and surgeons from cross-infection [5]. Negative pressure operating room was proposed during 2003 Severe Acute Respiratory Syndrome (SARS) pandemics, which was suggested as an alternative and enhanced protection to surgical staffs, as it provided a satisfactory airborne precaution [6]. One out of 17 operating rooms in the main block was a negative pressure theatre, and it was the priority choice for all emergent surgeries without RT-PCR nucleic acid tests. Regular positive pressure theatres were available for elective surgeries, with full personal protection equipped for all staff.

### Workflow

**Fig. 4:**
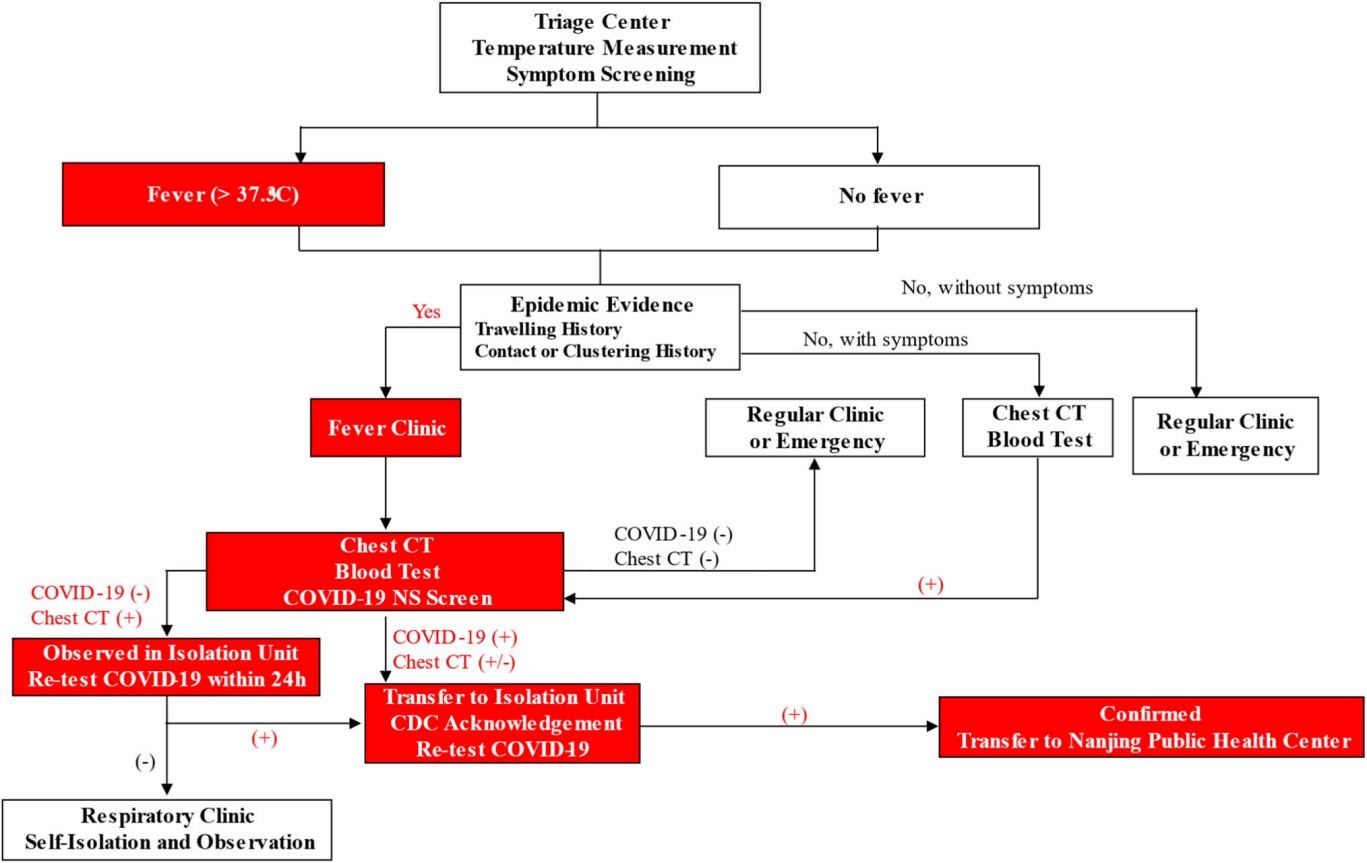
The flowchart showing the screening procedure in the outpatient clinic

Workflow for Outpatient Service—As shown in Fig. 4, patients with fever, respiratory symptoms or epidemic history would be transferred to the Fever Clinic. Chest CT, together with a blood test and Covid-19 nasopharyngeal swab (NS), was performed. Highly suspected patients with positive Covid-19 test and CT scan were further quarantined in the Isolation Unit for re-test by CDC. Double confirmed cases were immediately transferred to Nanjing Public Health Center, the only official hospital for Covid-19 treatment. MDT professionals were involved in evaluation and diagnosis.

**Fig. 5:**
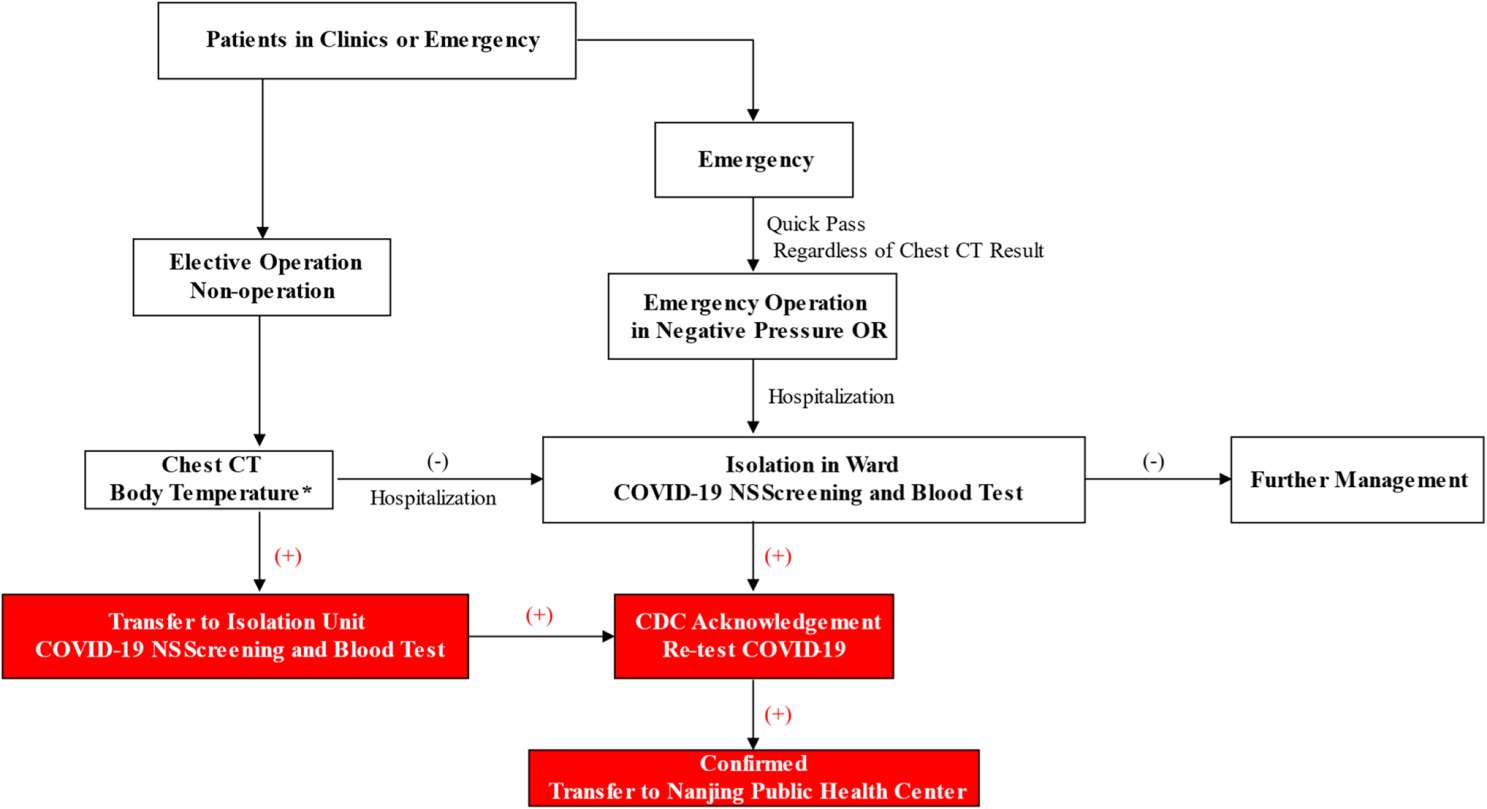
Screening procedures for hospitalization and surgery. * Special scenarios such as other infectious diseases causing fevers and CT abnormalities should be considered

Workflow for Hospitalization and Surgery Service—All in-patients went through the SARS-CoV-2 screening process to eliminate in-hospital transmission. Figure 5 Shows the screening process for hospitalization and surgery. In principle, only those with normal temperature and chest CT with negative findings were allowed for hospitalization. The notice should be taken for special scenarios such as other infectious diseases causing fevers and CT abnormalities. MDT, therefore, played an irreplaceable role in strategy making. Upon admission, patients were under individual isolation for compulsory virus screening. For those with SARS-CoV-2 positive, further management would be carried out upon CDC's re-examination.

A quick pass was applicable to emergent cases. Lacking evident exclusions, operations were allowed only in the negative pressure theatre. All surgeons and medical staffs were essentially equipped with a cap, goggles, N95 mask, shoe covers, and disposable surgical gown and single-use gloves. Special concerns arose in patients with microscopic surgeries like neurosurgeries because it is impracticable to operate under a surgical microscope with goggles or face shield. All medical wastes were collected in a SARS-CoV-2-labeled double-layer bag and handled with Clinical Waste Management Procedures. Decontamination procedures were then operated to the highest standard, and air within the theater was tested by IPC.

### Training on Prevention of Transmission of Covid-19

Training for Healthcare Providers—All staffs must finish certain courses to learn about the Clinical Practice Guidelines: National Guideline for Management of Novel Coronavirus Pneumonia Infections, and the standard operating procedures of our hospital on January 20. During Phase II, we applied online training sessions to ensure that healthcare providers updated knowledge of the latest national clinical practice guidelines and hospital emergency response plan. Several questionnaires concerned with the hospital's operational performance were designed to investigate staff satisfaction and knowledge acquirement. As the epidemic continues, due attention is paid to the physical, mental and emotional needs of doctors and nurses, and psychological intervention is available.

Training for non-medical personnel—Non-medical personnel in the hospital, including logistics, security and volunteers received the same level of personal protection from infection. They participated in skill training on hand hygiene, wearing isolation gowns, environmental disinfection, to enhance their ability to fulfill their roles in implementing emergency responses. Their regular exercises and activities were supervised by the IPC team.

Patient and visitor management—Patients and people who accompany them in general wards were required to wear (at least) surgical masks. Similar risk assessment of visitors was taken by senior nurses, and visitors from Hubei Province were forbidden. Only one single visitor at a time wearing a mask was allowed to visit a patient.

### Human Resource

Team of human resource is in charge of arrangement and assignment of medical care providers. We faced a shortage in medical personnel throughout the time not only nationally but also in our hospital for two reasons. First, being less experienced, all the interns and junior residents were not called back and under strict self-isolation at home due to the Chinese New Year holiday. Second, twenty physicians and nurses were sent to participate in the medical team supporting Wuhan and Hubei Province, while additional medical team with 150 physicians and nurses being standby, which might further worsen the situation of staff shortage. Under such circumstances, the Human Resources took several measures. First, volunteers from the community and administrative staffs were assigned to assist the temperature measurement, epidemic history inquiry and triage the patients at the entry of the outpatient clinic. Right-on-time education and training were provided to the above personnel to ensure their qualifications during the first phase of preparedness. Second, some retired hospital staffs were included in the personnel reserve to assist clinical care when necessary. Each staff's health condition was strictly recorded. Third, psychological support to relieve the anxiety among staff was available.

### Researches

To better understand and control the epidemics, several clinical trials were designed, registered and started. A randomized controlled trial was proposed by critical care medicine physicians of our hospital working in Wuhan, evaluating the safety and efficiency of a certain antiviral drug. Moreover, a large number of grants supported by various foundations such as National Natural and Science Foundation, local government, the university and hospital foundations were available to encourage more scientific researches on SARS-CoV-2.

### Information Technology Application

Online Consultation and Prescription system—As a supplement to outpatient service, a free online consultation and prescription system was opened for all the specialties to reduce the cross-infection risk and decrease the workload for medical staff. The system supports real-time and appointed photo/video consultation, while payment could be accomplished online. The working site is a dedicated meeting room equipped with developed information and network communication technology.

Daily Data Report—Daily operational statistics, including outpatient numbers, suspected/confirmed cases, personnel, resources and material reserve, were all uploaded to the Office Automation system for authorized access. The management policy and procedure of the epidemics were dynamically updated and shared promptly. In addition, advices and suggestions from employees can be conveniently collected through mobile and analyzed by the committee.

Online Education and Meeting—To avoid gathering, staff training for SARS-CoV-2, weekly meetings for administrators, case discussions and lecturers were all carried out through live streaming on a mobile device.

Remote Union Consultation Platform—In response to the ongoing epidemic in Hubei Province, Zhongda Hospital joined a remote union consultation platform, which connects seven provincial-level hospitals with more than 100 experts designated for diagnosis and treatment of Covid-19. Through the platform, experts could conduct image reading and report writing, case discussion, and develop a personalized treatment plan.

## Results

### Hospital-Acquired Covid-19 Infection

Until March 4, 3879 CT scans and 2305 PCR tests were accomplished, of which 0.4% revealed abnormal image patterns and 0.1% PCR positive, distinguishing 16 suspected patients and 3 confirmed cases. No medical care provider was infected with Covid-19. There is also no case of hospital-acquired Covid-19 infection for those who received medical care in the setting of both outpatient and inpatient.

### Interim Admission

**Fig. 6:**
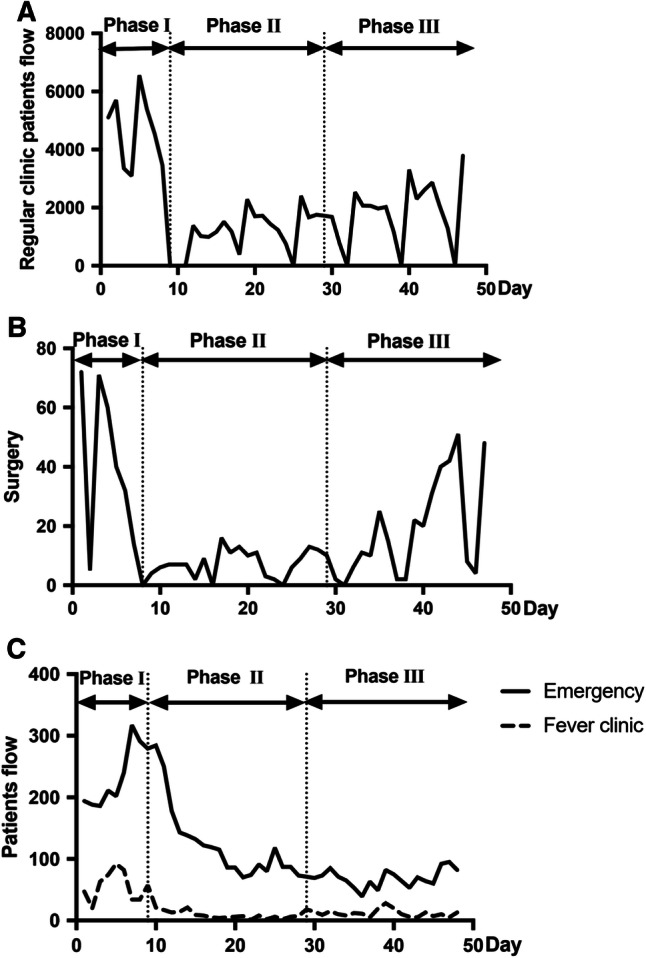
Interim admission during the epidemic. **A** Regular clinical services were available in Phase I and decreased due to the clinical restrictions in Phase II, then gradually increased in Phase III. **B** Surgeries maintained in Phase I, while urgent surgeries continued throughout three phases. **C** Patient volume reached a high level in Emergency (day 7, January 22) and Fever Clinic (day 5, January 20) during Phase I and gradually decreased in Phases II and III. In A and B, the curve reached bottom several times during Phases I and II. This was caused by the shut down of the regular clinic and surgery during weekends

With the outbreak of Covid-19, daily reports consist of outpatients, surgeries, fever clinics and emergencies were displayed to assess the flow during the epidemics. Regular clinical services (Fig. 6A) and surgeries (Fig. 6B) maintained in Phase I had a remarkable decrease due to the clinical and elective surgery restrictions in Phase II and gradually increased in Phase III. Notably, emergencies and fever clinics were faced with a prominent peak rapidly in Phase I, with 317 patients in emergency (day 7, January 22) and 92 patients in the Fever Clinic (day 5, January 20). Considering the public panic in this period, additional health educations were provided by doctors and nurses to relieve patients' anxiety, which might contribute to the gradual decrease in patients in Fever Clinic and emergency in Phase II and even in Phase III when work resumption began (Fig. 6C).

### Psychosocial Distress of Hospital Staff

Hospital staff may be particularly vulnerable to psychosocial distress as they faced a conflict between professional responsibilities and families. Questionnaires were designed to identify hospital staff members at high risk of suffering mental distress and other mental problems in the fight with Covid-19. Of all the valid questionnaires. 6.46% staffs suffered depression, and 9.87% had anxiety.

### Staff’s Feedback for Hospital Response on Covid-19

In addition, our infection control work was introspected through questionnaires to staff to feedback the suggestions and satisfaction. A comprehensive understanding of this novel coronavirus was displayed after training, with a scale of 95/100. Overall, most staff members (98%) were satisfied with the hospital infection control work, and excellent suggestions were provided.

### The Verification of the Donation and Daily Consumption

**Fig. 7:**
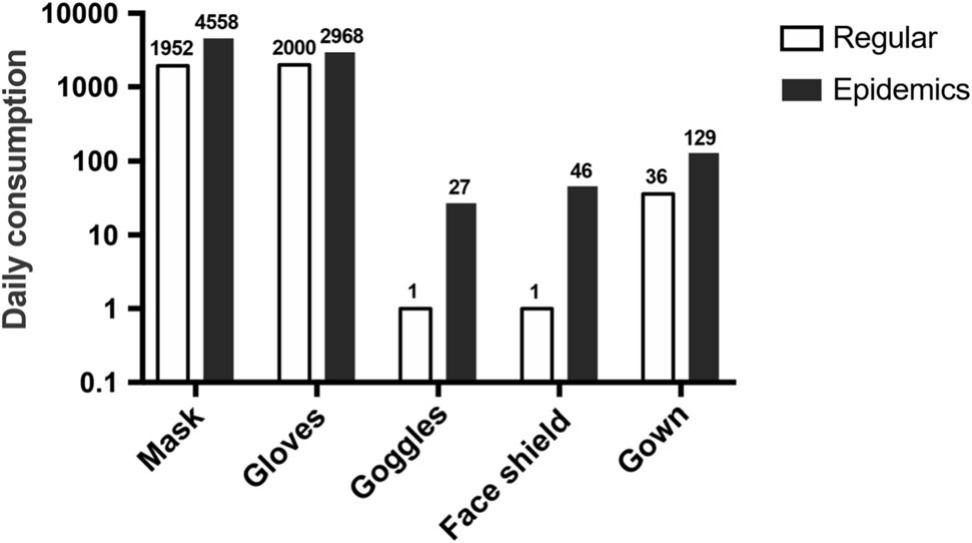
The comparison of the daily PPE consumption between epidemic and regular periods in a year-over-year manner. The increased demands for all categories were apparent

Our hospital has received a total of 315,009 masks, 13,579 gowns, 125,800 gloves, 6448 goggles and 3404 face shields through public donation by March 2. Daily consumption of PPE dramatically increased, and the requirements for masks and gowns were 2.3 and 3.6 times that of last year, respectively (Fig. 7). Additional demands for goggles and face shields highlight the necessity of routine store in case of such emergency.

## Discussion

At present, the risk of Covid-19 pandemic is higher than ever before. The containment of the uncontrollable outbreak has become a global priority in both developing and developed entities. This paper described our efforts in responding, organizing and managing hospital operation to prevent in-hospital transmission and infection. Our study documented no hospital-acquired Covid-19 infection during this epidemic period, indicating that the prompt and organized response plays an important role in infection prevention and control. Experience and obstacles are shared and discussed to assist strategy tailoring in other nations fighting against the virus.

Despite an extensive spectrum of studies and data illustrating Covid-19's epidemiological, biological, radiological and clinical features, great uncertainties and controversies still remain. With an ongoing worldwide dissemination, it is highly likely that all human races are vulnerable to this novel coronavirus. The transmission mode is still unclear, as the presence of SARS-CoV-2 in the blood and the feces indicates a potential multiple transmission pattern [7]. In a Wuhan Hospital, up to 29% hospital-associated infections among medical professionals were documented [8]. It alerts that uncontrollable transmission in medical facilities not only worsens personnel shortage, but more importantly potentiates virus spread.

Because neither vaccine nor particular treatments proved effective so far, excessive screening practice becomes the first-line measure to prevent cross-infection. At present, the screening of SARS-CoV-2 heavily relies on the RT-PCR of virus’ nuclei acid and ultra-resolution chest CT. However, the sensitivity of RT-PCR was reported barely 30–60% at the initial test [9]. As several relapsed cases with repeated negative PCR results were reported in China and Japan, its reliability is increasingly challenged. Difficulties in sample collections and transportations and the shortage in test kit supply further limited its large-scale application. However, routine application of chest CT is also questionable due to the excessive radiation exposure safety issue. Due to the presence of RT-PCR(−) /CT( +) cases [10] and the update of the Chinese CDC’s Clinical Practice Guidelines, chest CT is opted as an essential screening tool in our hospital.

Confronting the outbreak and the ever-changing situation, especially when coincided with the Chinese New Year holiday, our management strategies are dynamic and adapted to the updated knowledge of the virus and the epidemic evolvement in different phases. In the past nine weeks, Zhongda Hospital leveraged its administrative strengths and started an all-out war against the novel coronavirus epidemic. Together with the MDT and IPC Team, supported by the Supporting Team and Media Team, the Committee implemented the overall the hospital's management covering risk assessment, prevention and preparedness. As a comprehensive and busy hospital located in downtown area, our space for physical plant is limited and the infrastructure could not guarantee the standard quarantine. Therefore, a backup plan for quarantine unit was developed in case of large infections in Nanjing, which turned out to be effective. Suffering from the severe shortage of medical supplies, we accepted rapid and adequate public donations of PPE, oxygen supplying equipment and other hygiene consumables, which proved the active community participation.

We have to admit that, although we have been plagued by SARS, a highly infectious diseases caused by coronavirus in 2003, guidelines and protocols for pandemics management were still not readily available. To properly prevent in-hospital infection, we have to remodel regular units indicating that a specific quarantine unit must be considered and included in a comprehensive hospital. Negative pressure airflows are necessary when constructing the isolation unit, operating theater, Fever Clinic, etc.

Although the Covid-19 pandemic seems all but inevitable, there is still uncertainty about its severity worldwide. While time will tell eventually, hospitals should not delay. In the event of a pandemic, the predictable costs of not preparing, in human, societal and political terms, would be huge. Decision makers at all levels, including administrators and physicians, should consider how to proceed these issues. Several of the priority items (setting up administrative teams, allocation of scarce resources and carrying out education and training) take substantial time. Therefore, hospitals should begin to take actions as soon as possible.

We also noted that about 6·46% staffs suffered depression, and 9·87% had anxiety during this epidemic period. Supportiveness and intervention were provided and available to all hospital staff when necessary. Further efforts should be put into this area to decrease and prevent new onset of depression or anxiety.

Until now, with the efforts of both government and hospitals, the Covid-19 spreading in Nanjing is likely under good control and not as severe as in Wuhan. Otherwise, Zhongda Hospital would encounter many more challenges. Nevertheless, we believe that Zhongda would not have survived this battle without implementing the above-mentioned measurements. We have much more confidence to eventually conquer Covid-19 in the near future.

## References

[1] Zhu N, Zhang D, Wang W (2020). A novel coronavirus from patients with pneumonia in China, 2019. N Engl J Med.

[2] Gorbalenya AE, Baker SC, Baric RS, et al. The species Severe acute respiratory syndrome-related coronavirus: classifying 2019-nCoV and naming it SARS-CoV-2. Nat Microbiol. 2020 (**Epub ahead of print**) 10.1038/s41564-020-0695-z PMC7095448 32123347

[3] Organization WH. Coronavirus disease 2019 (COVID-19) situation report—59. https://www.who.int/docs/default-source/coronaviruse/situation-reports/20200319-sitrep-59-covid-19.pdf?sfvrsn=c3dcdef9_2. Accessed 20 March 2020.

[4] Organization WH. Laboratory Biosafety Manual. 2004. https://www.who.int/medical_devices/publications/lab_biosafety_manual/en/. Accessed Jan 16 2020.

[5] Seto WH, Tsang D, Yung RW (2003). Effectiveness of precautions against droplets and contact in prevention of nosocomial transmission of severe acute respiratory syndrome (SARS). Lancet.

[6] Chow TT, Kwan A, Lin Z, Bai W (2006). Conversion of operating theatre from positive to negative pressure environment. J Hosp Infect.

[7] Zhang W, Du RH, Li B (2020). Molecular and serological investigation of 2019-nCoV infected patients: implication of multiple shedding routes. Emerg Microbes Infect.

[8] Wang D, Hu B, Hu C, et al. Clinical characteristics of 138 hospitalized patients with 2019 novel coronavirus-infected pneumonia in Wuhan, China. JAMA. 2020 (**Epub ahead of print**) 10.1001/jama.2020.1585 PMC7042881 32031570

[9] Yang Y, Yang M, Shen C, et al. Evaluating the accuracy of different respiratory specimens in the laboratory diagnosis and monitoring the viral shedding of 2019-nCoV infections. medRxiv. 2020:2020.02.11.20021493.

[10] Ai T, Yang Z, Hou H (2020). Correlation of chest CT and RT-PCR testing in coronavirus disease 2019 (COVID-19) in China: a report of 1014 cases. Radiology.

